# Supplementary material for: Comparative multiomics analysis of cell physiological state after culture in a basket bioreactor
Source: Sci Rep. 2022 Nov 23;12:20161. doi: 10.1038/s41598-022-24687-4 (PMC9686226; doi:10.1038/s41598-022-24687-4)
Supplement: Supplementary file 1 — Supplementary Information 1. [file 41598_2022_24687_MOESM1_ESM.zip › raw data/Proteomic raw data/6.KEGGenrichment/EG.vs.CG/DOWN/pathway/src/04976.html]

map04976


- K09864

- Down regulated proteins

A0A0D9RQJ5(Description:Aquaporin-1;Gene:;log2FC:-1.4519)

- K09864

- Down regulated proteins

A0A0D9RQJ5(Description:Aquaporin-1;Gene:;log2FC:-1.4519)

Close
